# Analysis and best parameters selection for person recognition based on gait model using CNN algorithm and image augmentation

**DOI:** 10.1186/s40537-020-00387-6

**Published:** 2021-01-03

**Authors:** Abeer Mohsin Saleh, Talal Hamoud

**Affiliations:** 1 Damascus University, Damascus, Syria; 2 Syrian Private University, Damascus, Syria

**Keywords:** Person recognition, Convolution neural network, Gait model, Deep learning, Image augmentation

## Abstract

Person Recognition based on Gait Model (PRGM) and motion features is are indeed a challenging and novel task due to their usages and to the critical issues of human pose variation, human body occlusion, camera view variation, etc. In this project, a deep convolution neural network (CNN) was modified and adapted for person recognition with Image Augmentation (IA) technique depending on gait features. Adaptation aims to get best values for CNN parameters to get best CNN model. In Addition to the CNN parameters Adaptation, the design of CNN model itself was adapted to get best model structure; Adaptation in the design was affected the type, the number of layers in CNN and normalization between them. After choosing best parameters and best design, Image augmentation was used to increase the size of train dataset with many copies of the image to boost the number of different images that will be used to train Deep learning algorithms. The tests were achieved using known dataset (Market dataset). The dataset contains sequential pictures of people in different gait status. The image in CNN model as matrix is extracted to many images or matrices by the convolution, so dataset size may be bigger by hundred times to make the problem a big data issue. In this project, results show that adaptation has improved the accuracy of person recognition using gait model comparing to model without adaptation. In addition, dataset contains images of person carrying things. IA technique improved the model to be robust to some variations such as image dimensions (quality and resolution), rotations and carried things by persons. Results for 200 persons recognition, validation accuracy was about 82% without IA and 96.23 with IA. For 800 persons recognition, validation accuracy was 93.62% without IA.

## Introduction

Gait is a kind of behavioral biometric feature, it is defined as the way a person moves and the movement of every person has no typical form or scenarios.

There are other behavioral biometrics like face and iris but they are limited by the distance between the person and the used camera. There are two types of biometric features soft and hard; the hard ones are also considered classic or traditional, such as faces, fingerprints or signatures. The soft ones are related to faces, as skin color, hair color or facial measurements, other soft are related to bodies, like height or weight, and to accessories, such as glasses or hats [1]. The unique characteristics of gait, such as unobtrusive, non-contactable and non-invasive, have a powerful potential to apply in the scenarios including criminal investigation, access security and surveillance [2]. In general, gait can be used for the human recognition task. Not only person recognition can be estimated by gait, gender and age can also be estimated by it [3].

In addition, PRGM has a many application in real time life, it offers a useful tool for non-invasive biometric validation, and human-robot interaction in a broad range of applications from crowd traffic management to personalized health care. Considering the gait as a set of poses and movements, gait information can be extracted from images or videos from static cameras, the information represent the gait of person [4].

PRGM also has important role in video surveillance. It is particularly challenging because observed pedestrians undergo significant variations across camera views, and there are a large number of pedestrians to be distinguished given small pedestrian images from surveillance videos [5].

Some researches work on person recognition and assume that observations of persons are captured in relatively short periods, such that clothes and body shapes do not change much and can be used as cues to recognize identity. In video stream surveillance, the captured persons are often small in size, facial or iris features are indistinguishable in images and face or iris recognition techniques are not applicable. Therefore person recognition techniques with gait models become important [6].

There are two ways to get gait features for any person cameras and sensors. Surveillance cameras may observe tens of thousands pedestrians by images in a public area in one day and many of them look similar in appearance. cameras images might be small images and contain sometimes noise or it could be blurry image, so we need a powerful tool to to be robust to these conditions. Our designed model treat person recognition regardless the large variations of lightings, poses, viewpoints, blurring effects, image resolutions, camera settings, and background across camera views, the designed model has many enhancements as we will see.

Other method for gait model is recording using sensors; motion sensors are available widely such as accelerometers and gyros, these sensors are located on the human body; in which gait analysis is performed based on the readings of the sensors, sensors might be built into the smart-phones. In this regard, especially important is the problem of recognizing the person using his gait [7].

Using sensors (as mentioned above) might be undesirable by the person, cameras can be captures images or videos of person without the attention of person, so using cameras are suitable for gait analysis and person recognition. Images from cameras differs in some issues according to some conditions:

- Cameras resolution.
- Cameras position.
- Weather conditions (night, day, light issues).

To handle these conditions, we need a powerful tool, deep learning tool (CNN as example) to deal with all condition and search deeply to find all possible features for each person. After mentioning many application for PRGM and with the increasing demand for person recognition in the era of big data and artificial intelligence, the research and development of many algorithms attracted broad attention from both academia and industry, e.g., the fingerprint, iris, face, and voice etc., have been implemented commercially. All examples in this field will output huge amount of data ( 1,2 or dimensions), therefore we need a Convolution neural network to handle features that can be concluded from the input data.

In addition to data in our hand; the persons images, we also worked on image augmentation and thus will increase the amount of data multi times. So this research addresses the problem of person recognition depending on gait model with main research points:

- Best selection of both parameters and the design of CNN
- Using Image Augmentation to increase person features and to make trained model robust to some variations in the image
- Understand the structure, layers and parameters of CNN to implement it and be able to understand how to change CNN properties.

## Background and related work

In [8], authors introduce a simulation-based methodology and a subject-specific dataset to for generate synthetic video frames and sequences for data augmentation to help in gait recognition. They generated a multi-modal dataset. In addition, they supply simulation files that provide the ability to simultaneously sample from several confounding variables; these variables are about brightness, rotation, color saturation. The basis of the data is real motion capture data of subjects walking and running on a treadmill at different speeds. Results from gait recognition experiments suggest that information about the identity of subjects is retained within synthetically generated examples. This study has applied image augmentation using render program to generate an accurate sequence of motion as real as possible, this is costing processing time, they also did not consider body form (fat, this, short and tall).

In [9], the authors used a virtual environment, which enabled them to present the same type of gait across different identities. Using this setting, they assessed the accuracy and distance at which identities are recognized based on their gait, as a function of gait distinctiveness. Furthermore,the virtual environment also enabled them to assess. They find that the accuracy and distance at which people were recognized increased with gait distinctiveness. Overall these findings highlight an important role for gait in real life person recognition and stress that gait contributes to recognition independently from the face and body. The virtual environment helped them a lot in their research.

In [10], authors proposed a gait recognition approach for person re-identification. The proposed approach starts with estimating the angle of gait first, and this is then followed with the recognition process, which is performed using convolution neural networks. Here in, multi-task convolution neural network models and extracted Gait Energy Images (GEI) are used to estimate the angle and recognize the gait. GEIs are extracted by first detecting the moving objects, using background subtraction techniques. The proposed gait recognition method showed an accuracy of more than 98% for almost all used datasets. The authors worked on GEIs, there is a feature engineering process before re-identification process.

In [4], the authors devoted to the problem of the recognition of a person by gait using a video recorded in the optical range depending on detection of a moving person on a video sequence with the subsequent size normalization and dimension reduction using the principal component analysis (PCA) technique. The person classification was carried out using the support vector machine (SVM). Authors have determined the best values of the method parameters; their method for person recognition has next steps: detection and segmentation of a moving person in the video sequence, normalization of the frame size of the selected video sequence fragment, dimensionality reduction of the selected video sequence fragment and classification of video sequences. The obtained results showed high classification accuracy with small number classes.

In [11], The authors have studied recognition performance when handling confounding variables, such as clothing, carrying and view angle. A novel method was proposed to explicitly disentangle pose and appearance features from RGB imagery to get pose features over time produces. In addition, authors focused on gait recognition from frontal-view walking, which is a challenging problem since it contains minimal gait cues compared to other views. The method demonstrated superior performance compared with other researches. The cases of tests focused on rotations and variations from only front view walking.

In [12], The author has applied a gait recognition depending on the reality that every human has a distinctive walking style; which is proposed to be used in gait recognition as an identification criterion, Author has applied CNN with help of center-of-pressure (COP) trajectory that is sufficiently unique to identify a person with high certainty. Using a platform to record COP for a period then using these records to classify only 30 persons, this method requires a platform for each person so it is expensive.

In [13],The authors developed a specialized deep CNN architecture for Gait Recognition. The proposed architecture is less sensitive to several cases of the common variations and occlusions that affect and degrade gait recognition performance. It can also handle relatively small data sets without using any augmentation or fine-tuning techniques. The majority of previous approaches to gait recognition have used subspace learning methods which have several shortcomings that we avoid. Their specialized deep CNN model can obtain competitive performance when tested on the CASIA-B large gait data set; CASIA data set has only 20 persons.

The usage of neural network (the basic thing in CNN model) has increased remarkably lately, Many studies on neural networks in new fields is are rising every day. In Compacting Concrete as in [14], In navigation fields as in [15]. In quantum physics as in [16].

In this study, the tests were on data set that has images for 200 persons, most of the studies tested on data sets with small number of persons. In addition, the data set images have low resolution, only 64*128. Some studies depend on ready models with some modification or tuning. In this study, CNN model is built step by step as it will be explained in next paragraphs.

The main benefit of our study can be summarized in two main points:

*First point*: Image augmentation for gait recognition no matter what used algorithm was. In our case, CNN was used.

*Second point*:Using CNN algorithm and best selection for:

- CNN parameters.
- CNN design and structure.

## Objective of the paper

**Fig. 1:**
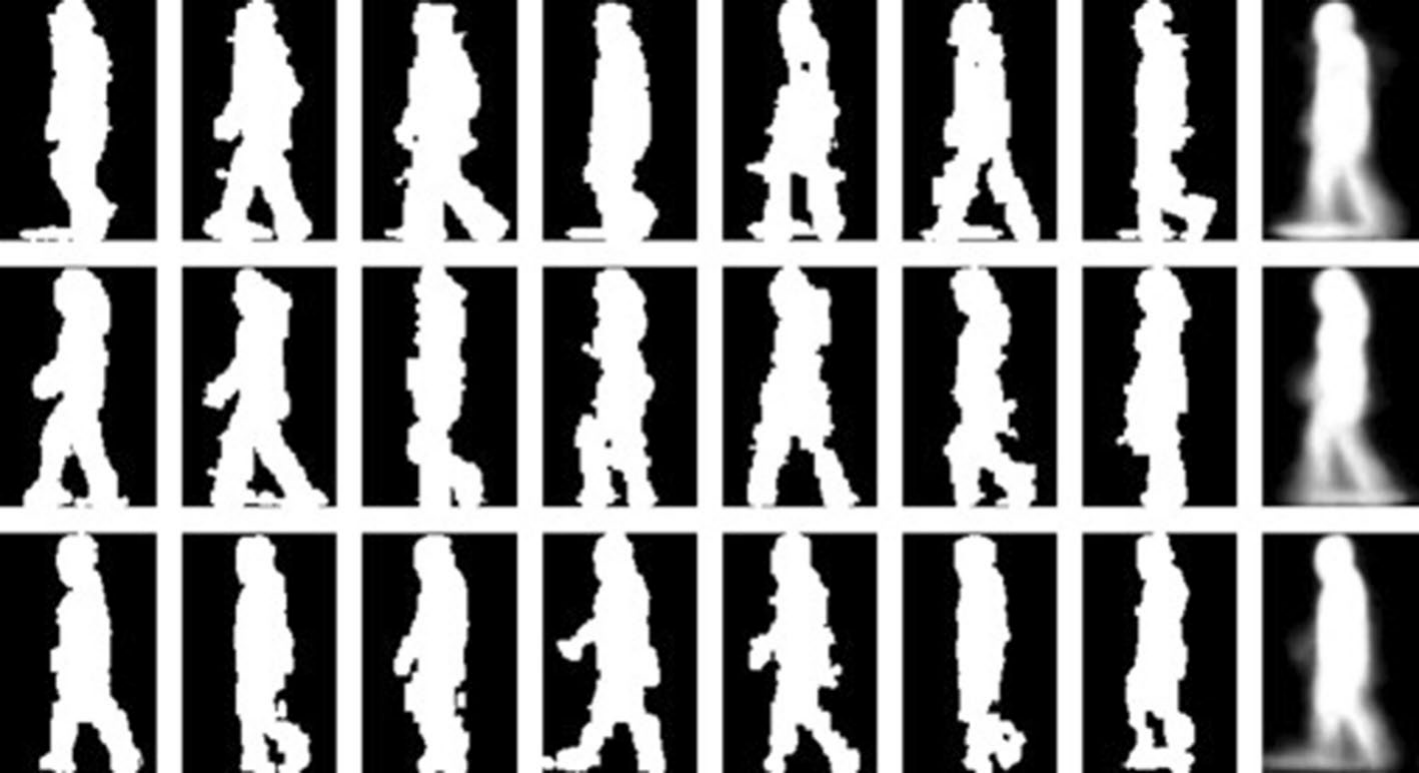
Example of Gait model after image processing

Actually, the main intention of this work in general is to master the person recognition based on gait features and improve the recognition as possible as we can. The other goal is to apply improved recognition system in application such as tracking COVID spread and recognition people in cases where there is no fingerprint, no iris detection and no clear view of the face. Examples of Gait model is presented in the Fig. 1 below [17]

In practice, gait model recognition is often used when cameras are installed around the target subjects (persons), which may be unaware of the observation for prisoners or criminals [5].

**Fig. 2:**
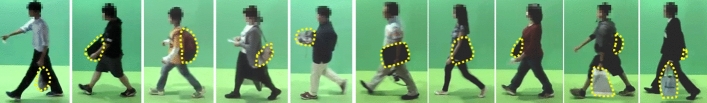
Gait model with carrying objects

New reports about gait models are collected for corona-virus states. Under this title “Corona-virus gives China more reason to employ biometric”, tablets (containing cameras) have recently been installed by the driver’s seat in public buses and in many places. Passengers are expected to put their foreheads close to the tablet so that their temperatures and photos can be taken. The photos taken by the cameras (more than 200 millions cameras) can then be used in person recognition depending on many terms including gait model that might be ill with Corona-Virus. The number of CCTV1 cameras in China in 2019 is 200 and it will be increase to 626 million by 2020 [18]. Gait model might have some carrying objects which is considered as noise in the image [19] as shown in Fig. 2.

According to the design of Convolution Neural Network, one image with dimension 100*100 may be during the CNN stages as thirty images with less dimension (supposed 50*50), so a data 10000 will convert to 75000 (7.5 times) for one image. for a dataset of 5000 images, data during CNN may be about 375000000 that is considered as big data issue. The term “big data” refers to data that is so large, fast or complex that it is difficult or impossible to process using traditional methods.

Image augmentation is one useful way for building deep learning algorithm that can increase the size of the training set without acquiring new images. The idea is to duplicate images with some kind of transformations so the model can learn from more examples. Ideally, image can be augmented in a way that preserves the features key to making predictions, but rearranges the pixels enough that it adds some noise. Augmentation will be counterproductive if it produces images very dissimilar to what the model will be tested on, so this process must be executed with care [20].

## Methods

Convolution Neural network model is an important type of feed-forward neural network with special success on applications where the target information can be represented by a hierarchy of local features [21]. A CNN is defined as the composition of several convolution layers and several fully connected layers [22]; it is helpful tool for recognizing people through their gaits; neural networks analysis gait model to extract multiple complex features. Convolution neural networks (CNNs) have been used with great success for video-based gait recognition. CNN is well used in BigData field with many application, that image is extracted many times through the CNN process [23].

CNNs are especially well suited for working with images as a result of their strong spatial dependencies in local regions and a substantial degree of translation invariance. Similarly, time series can exhibit locally correlated points that are invariant with time shifts. The successful use of deep CNNs for the classification of unidimensional or multidimensional time series has been attested [24]. Like for image classification, CNNs can extract deep features from a signal’s internal structure. CNNs are potent tools for bypassing feature engineering in signal processing tasks (end-to-end learning) [25]. However, CNNs, like other artificial neural networks, require hundreds of examples in each class for efficient learning; therefore, they have not been applied in footstep recognition studies so far due to the difficulty of collecting many strides using sensing floors or force platforms.

There are a lot of algorithms that people used for recognition problems which their input is images before CNN became popular. In general, we need to create features from images and then feed those features into machine learning algorithm like SVM. Some algorithm also used the pixel level values of images as a feature vector too. As an example, SVM could be trained with 784 features where each feature is the pixel value for a 28 × 28 image.

CNN work so much better

- CNNs can be thought of automatic feature extractors from the image. While if I use a algorithm with pixel vector I lose a lot of spatial interaction between pixels; feature engineering is not required in CNN [26].
- CNN effectively uses adjacent pixel information to effectively down-sample the image first by convolution and then uses a prediction layer at the end[27].
- CNN includes the multiple uses of the convolution operator in image processing.
- The CNN architecture implicitly combines the benefits obtained by a standard neural network training with the convolution operation to efficiently handling the requested tasks; classification, recognition and identification.
- CNN is also scalable for large datasets.

For this study, the typical deep CNN is consisted of [28]:

- Input
- Convolution layer
- Pooling Layer
- Fully connected layer (FC)
- Output

**Fig. 3:**
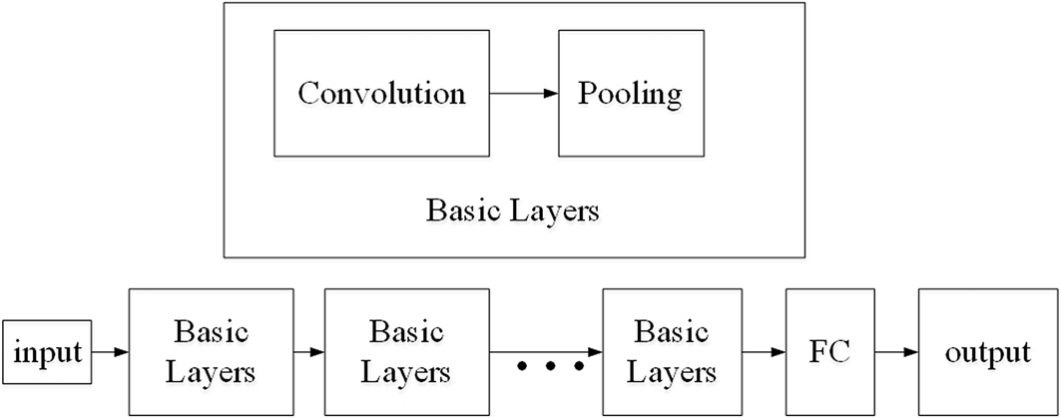
Basic CNN Design

Basic CNN design is shown in Fig. 3

Each part has its own parameters and properties, convolution layer has:

- nb filter: The number of convolution filters.
- filter size: Size of filters (kernal size).
- Strides: Strides of convolution operation.
- Padding: is simply a process of adding layers of zeros to input images to avoid problem related to size after convolution process.
- Activation: Activation function applied to this layer (Default is linear).
- Bias: If True, a bias is used.
- Weights init: Weights initialization.
- Trainable: If True, weights will be trainable.
- Regularizer: Regularization is commonly used for alleviating over-fitting in machine learning. For CNN, regularization methods, such as DropBlock and Shake-Shake, have illustrated the improvement in the generalization performance [29].

There are other parameters for pooling, regression and fully connected layer.

For Pooling layer:

- Kernal size: kernal size.
- Strides: Strides of pooling operation.
- Padding: Same as in convolution layer.

Fully connected layer has:

- Neurons
- Activation function

Regression Layer:

- Loss functions: are a key part of any machine learning model: they define an objective against which the performance of the model is measured.
- Learning Rate: helps to converge faster. Choosing a wrong learning rate, either too small or too large, can have a huge impact on the output.
- Optimizer: to minimize the provided loss function ’loss’ (which calculate the errors).

There is also a dropout that refers to dropping out units (both hidden and visible) in a neural network. Dropout refers to ignoring neurons during the training phase of certain set of neurons by a term named Keep Probability, which is chosen at random. By “ignoring”; i.e. these units are not considered during a particular forward or backward pass [30].

**Fig. 4:**
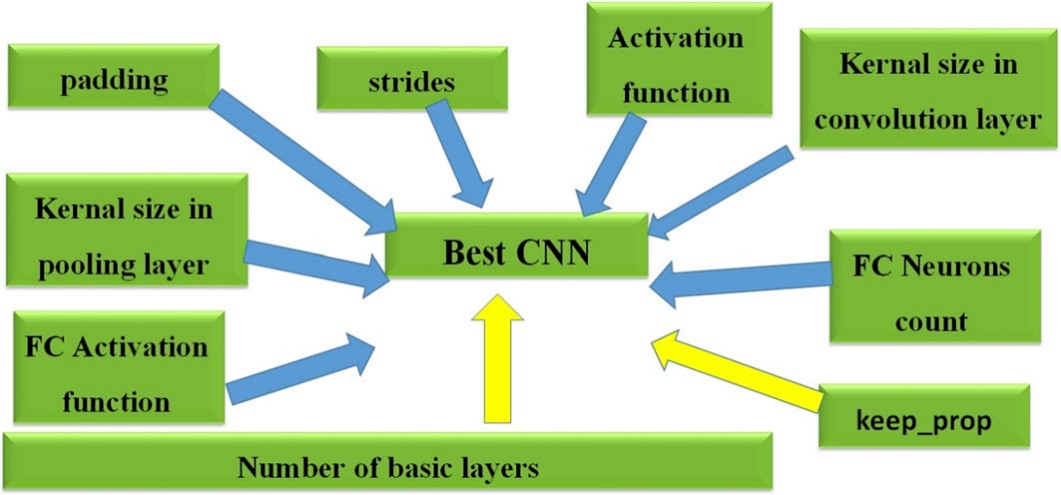
Best CNN parameters

The main work is to improve CNN depending on these parameters as shown in Fig. 4:

### Market dataset

#### Data set preparing

First of all, Data set was downloaded from [31], this dataset contains images that have been captured in front of supermarket for Tsinghua University:

- Six cameras (5 cameras with high resolution and one with low resolution).
- General environment (in front of supermarket).
- 1501 identity with more than 12900 images.
- Every person is existed in two images of two cameras at least.
- Image name contains:The person number, camera number, and image sequence number within the scene which is a random number Example 0001c1s100230100: person number 0001, camera c1, and image scene s1.

### Feature extraction

For convolution neural network, it automatically detects the important features without any human supervision. The convolution layer detects features such as head, long ears, legs, hands, trunk and so on. The fully connected layers then act as a classifier on top of these features. The sequence of images for the same person with will explored as temporal features for the person. There is no definition for gait imprint. somehow, we can say that gait imprint is a collection of features that represent body parts and their changes with time.

The convolution layers in CNN are the basic and important powerhouse of any CNN model. They automatically detect significant features. The convolution layers learn such complex features by building on top of each other. The first layers detect edges in the image, the next layers combine edges and lines to detect shapes or objects, to following layers merge this information to infer that this is a body or hands or legs etc. To be clear, the CNN is blind for these things; it does not know what a face is. By seeing a lot of them in images, it learns to detect that as a feature. The fully connected layers learn how to use these features produced by convolutions in order to correctly classify the images.

## Implementation and results

### Programming environment

**Fig. 5:**
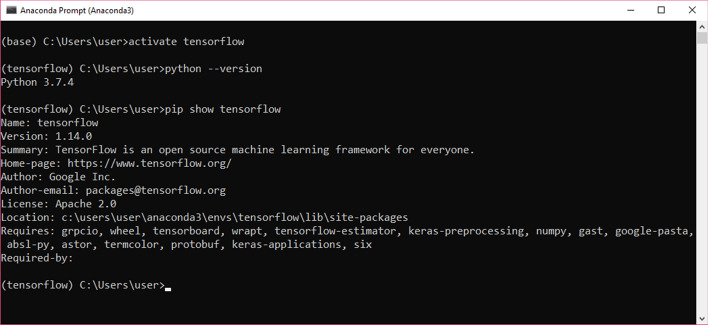
Python version

Anaconda was used as programming environment. According to many references, Anaconda is a free and open-source distribution of the Python and R programming languages for scientific computing (data science, machine learning applications, data processing, predictive analytics, etc.), that aims to simplify package management and deployment. The distribution includes data science packages suitable for Windows, Linux, and macOS. It is developed and maintained by Anaconda, Inc., which was founded by Peter Wang and Travis Oliphant in 2012. Details about python version is shown in Fig. 5.

With tensorflow version 1.14.0 and python version 3.7.4. TFlearn was used for implementation, Tflearn is a modular and transparent deep learning library built on top of Tensorflow. It was designed to provide a higher-level API to TensorFlow in order to facilitate and speed-up experimentations, while remaining fully transparent and compatible with it. Keras is also can be used, but Tflearn was preferred because of:

- TFLearn allows to use Python arrays directly.
- TFLearn allows to save models as checkpoint, index, and meta files, these files are used to create a frozen version models easily. Frozen models are very important to be used in Android apps or C++ programs.
- Keras saves models as HDF5 (The Hierarchical Data Format version 5) files, using which requires new skills again. Additionally, h5py library is need to be installed.
- TFLearn API is closer to that of TensorFlow.

### Model improvement

200 person were used from Market data set, 3104 images for training set and 840 images for testing set. For validation and testing, only 50 epochs were ran because training process might take too much time, all results in next paragraphs were for validation accuracy, since there are overall accuracy and validation accuracy. Model improvement was applied by testing many terms as follows to select best values of simulated terms.

Our main work is most like a genetic algorithm work. For each term we tested some values and monitored the accuracy and at which epoch the model reached high accuracy. In addition to that, IA was implemented to improve recognition accuracy. Edits on design and structure were done for best improvement.

#### Testing learning rate

First of all, Define next terms: CL: Convolution Layer, PL: Pooling Layer, FC: Fully Connected. Tests details are:

$$Input\rightarrow CL1\rightarrow PL1\rightarrow CL2\rightarrow PL2\rightarrow CL3\rightarrow PL3\rightarrow CL4\rightarrow PL4\rightarrow CL5\rightarrow PL5\rightarrow $$

$$FC1\rightarrow Dropout\rightarrow FC2\rightarrow Regression$$

CL1: 32 filters, Kernal size = 3, activation function: relu, bias:true

CL2: 64 filters, Kernal size = 3, activation function: relu, bias:true

CL3: 128 filters, Kernal size = 3, activation function: relu, bias:true

CL4: 64 filters, Kernal size = 3, activation function: relu, bias:true

CL5: 32 filters, Kernal size = 3, activation function: relu, bias:true

relu: Rectified linear unit function

PL1-2-3-4-5: Kernal size = 5, Pooling type: max

FC1: 1024 neurons, activation function: relu

FC2: number of persons for neurons counts, activation function: relu

Dropout: keep probability = 0.5

Regression:optimizer = ’adam’,loss =’categorical crossentropy’

by changing learning rate, and watching the validation accuracy: (Table 1)

**Table 1:** Results of changing LR

LR	Validation accuracy (%))
1e−5	Too bad
1e−4	37
5e−4	55
7e−4	58.45
8e−4	59.17
9e−4	59
1e−3	56
2e−3	44
5e−2	Bad

From this results, LR is 8e−4

Adam is an algorithm for first-order gradient-based optimization of stochastic objective functions, based on adaptive estimates of lower-order moments [32].

#### Testing dropout

Choosing LR = 8e−4, by changing learning dropout, and recording the validation accuracy (Table 2):

**Table 2:** Results of changing dropout

Dropout	Validation accuracy (%))
0.3	59.29
0.5	59.17
0.7	63.57
0.8	64.29
1	55.12

From this results, best dropout value is 0.8

#### Testing pooling Kernal size

Choosing LR = 8e-4, and dropout = 0.8, by changing Kernal size of pooling layer, and watching the validation accuracy (Table 3):

**Table 3:** Results of changing pooling kernal size

Dropout	Validation accuracy (%))
3	63.69
5	64.29

From results above, we can choose the value 5 to be the best Pooling Kernal size. From the tests, we have figured out that high kernal size will result in fast learning. There are two types of pooling MAX and AVERAGE, we tested two types but the average type was better than other.

#### Testing other parameters

Other parameters have not add any new improvements:

- Convolution layer kernal size: the value 3 was best for model.
- Padding and strides: keep the default values.
- loss function for regression layer: categorical cross-entropy since the labels are the Identities of persons and there are many labels (not binary issue).
- Adaptive Moment Estimation (Adam) optimizer works better (faster and more reliably reaching a global minimum) when minimizing the cost function in training [32].

### Model improvement with new design

**Fig. 6:**
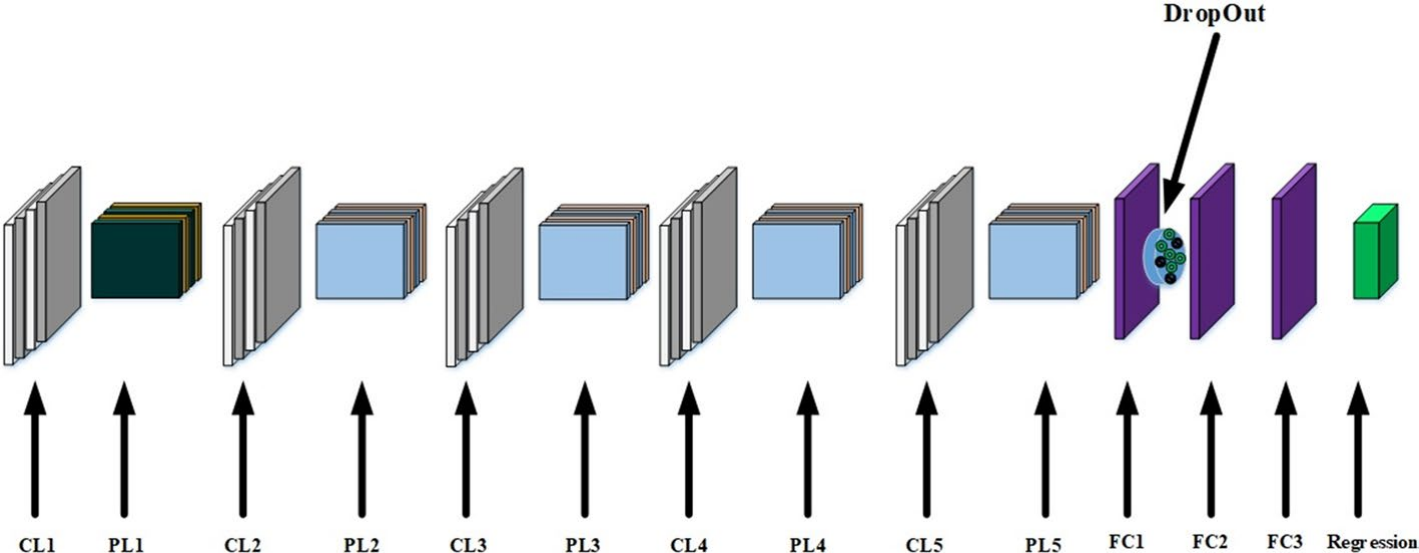
Proposed Design

After choosing some parameters, changes of design was adopted as shown in Fig. 6, these edits were implemented to improve accuracy:

$$Input\rightarrow CL1\rightarrow PL1\rightarrow CL2\rightarrow PL2\rightarrow CL3\rightarrow PL3\rightarrow CL4\rightarrow PL4\rightarrow CL5\rightarrow PL5\rightarrow $$

$$FC1\rightarrow Dropout\rightarrow FC2\rightarrow FC3\rightarrow Regression$$

CL1: 50 filters, Kernal size = 3, activation function: relu, bias:true, regularizer = ‘L2’

CL2: 100 filters, Kernal size = 3, activation function: relu, bias:true, regularizer = ‘L2’

CL3: 200 filters, Kernal size = 3, activation function: relu, bias:true, regularizer = ‘L2’

CL4: 100 filters, Kernal size = 3, activation function: relu, bias:true, regularizer = ‘L2’

CL5: 50 filters, Kernal size = 3, activation function: relu, bias:true, regularizer = ‘L2’

PL1: Kernal size = 5, Pooling type: max

PL2-3-4-5: Kernal size = 5, Pooling type: average

FC1: 4096 neurons, activation function: relu

FC2: 4096 neurons, activation function: relu

FC3: number of persons for neurons counts, activation function: relu

Dropout: keep probability = 0.8

Regression:optimizer = ’adam’,loss = ’categorical crossentropy’, Learning rate = 8e−4

**Fig. 7:**
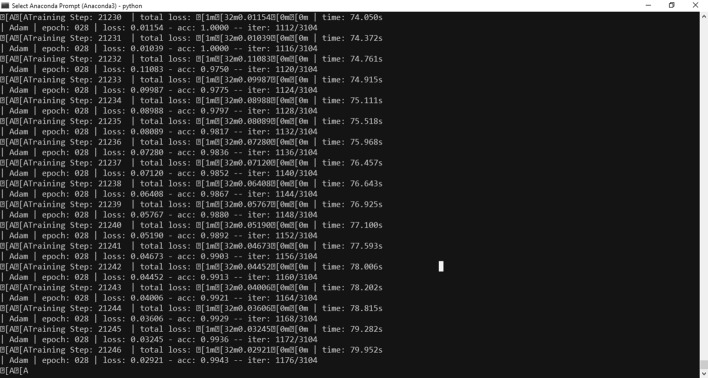
Improvement of updated design

Results are shown in the Fig. 7:

The overall accuracy has improved but still slow in converging. At epoch 28 has the accuracy about 99%.

In L2 regularization, the sum of all the parameters squared is calculated and added it with the square difference of the actual output and predictions, the value of the parameters will decrease as L2 will penalize the parameters.

#### Batch normalization

Batch normalization is a used technique to evolve the design of trained model in machine learning and improve the speed, performance, and stability of artificial neural networks. It is used to normalize the input layer by re-centering and rescaling [33]. batch normalization allows each layer of a network to learn by itself a little bit more independently of other layers. There are other normalization methods, local response normalization (LRN). LRN is a non-trainable layer that square-normalizes the pixel values in a feature map in a within a local neighborhood. Batch Normalization (BN): is a trainable layer normally used for addressing the issues of Internal Covariate Shift (ICF); it increases the stability of a neural network generally. ICF arises due to the changing distribution of the hidden neurons/activation. The output for normalization for some of pixels centered in X is calculated as follows where Y is the output.

1 $$\begin{aligned} \mu= & {} \frac{1}{m}{\sum _{i=1}^{m}{X_i}} \end{aligned}$$

2 $$\begin{aligned} \sigma= & {} \frac{1}{m}{\sum _{i=1}^{m}{{\left( X_i-\mu \right) }^2}} \end{aligned}$$

3 $$\begin{aligned} \hat{X}\,=\, & {} \frac{X-\mu }{\sqrt{\sigma ^2+\epsilon }} \end{aligned}$$

4 $$\begin{aligned} Y\,=\, & {} \lambda *\hat{X} +\beta \end{aligned}$$

$\lambda$ and $\beta$ are trainable parameters to get best performance.

**Fig. 8:**
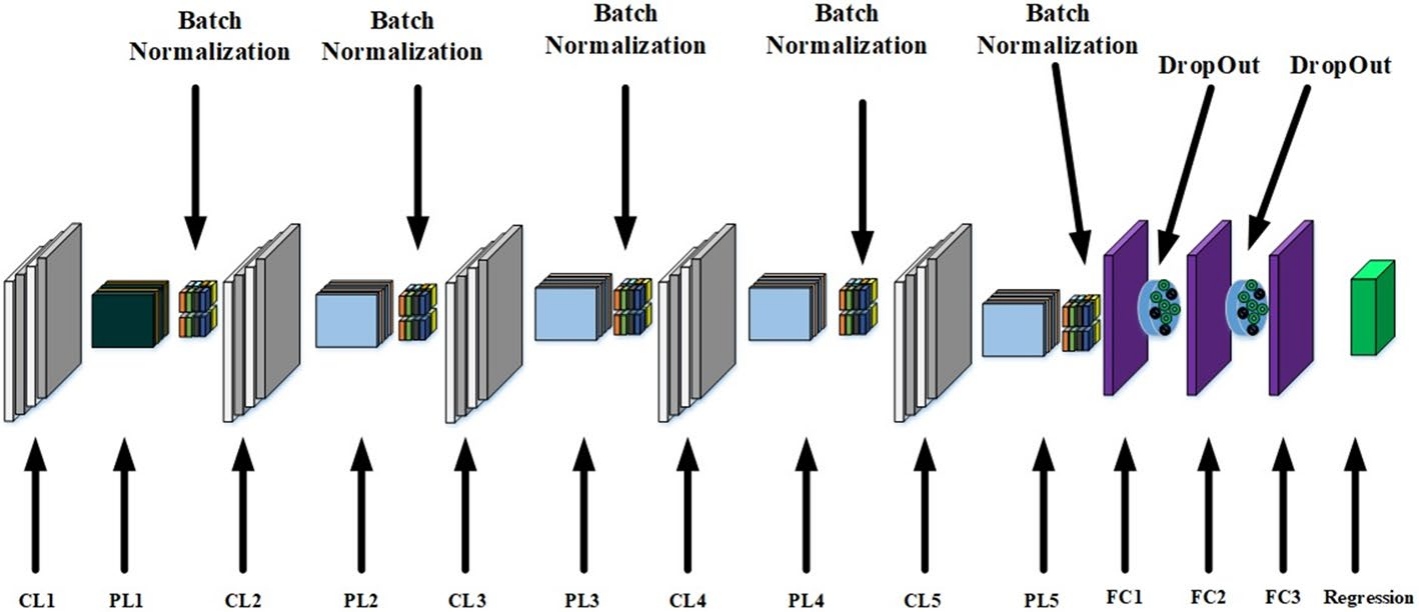
Results of updated design

The updated design is shown in Fig. 8: BM: stands for Batch Normalization

$$Input\rightarrow $$

$$CL1\rightarrow PL1\rightarrow BN1\rightarrow CL2\rightarrow PL2\rightarrow BN1\rightarrow $$

$$CL3\rightarrow PL3\rightarrow BN1\rightarrow CL4\rightarrow PL4\rightarrow BN1\rightarrow $$

$$CL5\rightarrow PL5\rightarrow BN1\rightarrow FC1\rightarrow Dropout\rightarrow FC2\rightarrow Dropout\rightarrow FC3\rightarrow Regression$$

CL1: 50 filters, Kernal size = 3, activation function: relu, bias:true, regularizer = ‘L2’

CL2: 100 filters, Kernal size = 3, activation function: relu, bias:true, regularizer = ‘L2’

CL3: 200 filters, Kernal size = 3, activation function: relu, bias:true, regularizer = ‘L2’

CL4: 100 filters, Kernal size = 3, activation function: relu, bias:true, regularizer = ‘L2’

CL5: 50 filters, Kernal size = 3, activation function: relu, bias:true, regularizer = ‘L2’

Without regularizing convolution layers, they may learn a over-fitted feature extraction which is not generalizable. It means that the features would be very distinctive for training set while they are not for the test set. If features are over-fitted, the model also may be over-fitted.

**Fig. 9:**
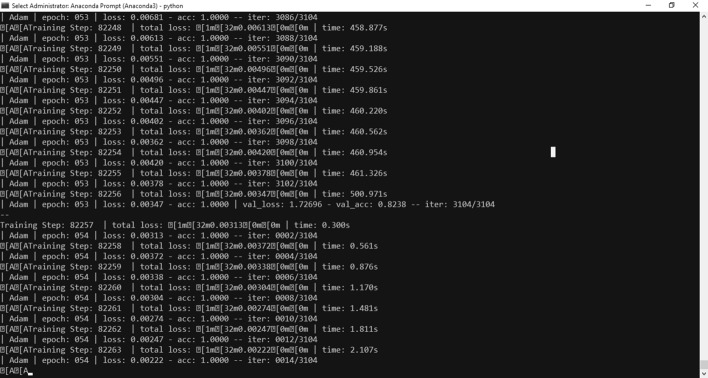
Final Results

**Fig. 10:**
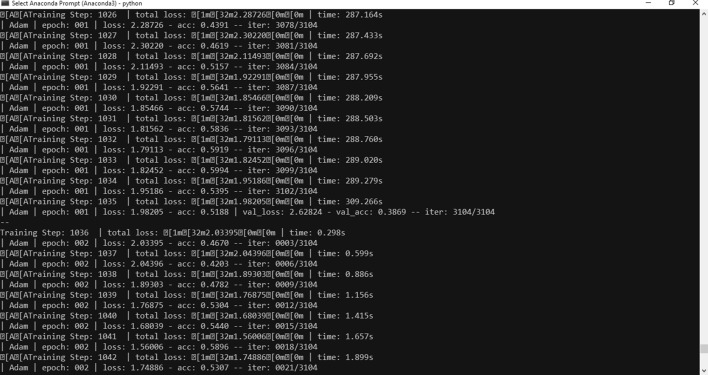
Speed is improved

The overall accuracy has improved very well to became 100 % at epoch 54 as shown in Fig. 9. and validation accuracy is about 82 %. Moreover, the speed of converging is increased as show in Fig. 10.

**Fig. 11:**
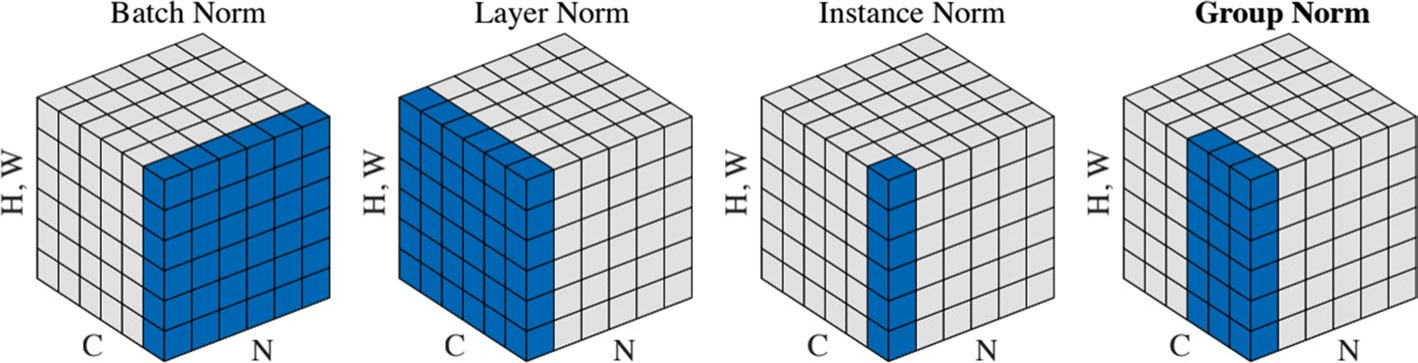
Batch explain

Figure 11 explains BN and other modes of Normalization for image (In convolution layers) with dimensions H: Height, W: Width [33] There are:

- Batch norm
- Layer norm
- Instance norm
- Group norm
- Weight norm
- Batch-Instance norm
- Switchable norm

### Image augmentation

Image augmentations have become a common implicit regularization technique to combat over-fitting in deep learning models and are ubiquitously used to improve performance. Common transformations that are typically used: flipping, rotating, scaling, and cropping. In our case, we used the next Image augmentation function (Table 4)

**Table 4:** Image augmentation

datagen = ImageDataGenerator(	
Rotation-range = 40,	
Shear-range = 0.2,	
Zoom-range = 0.2,	
Horizontal-flip = True,	
Brightness-range = (0.5, 1.5))

**Fig. 12:**
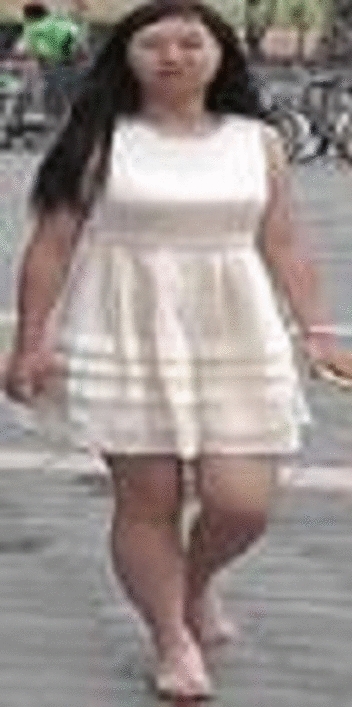
Simple image for augmentation

**Fig. 13:**
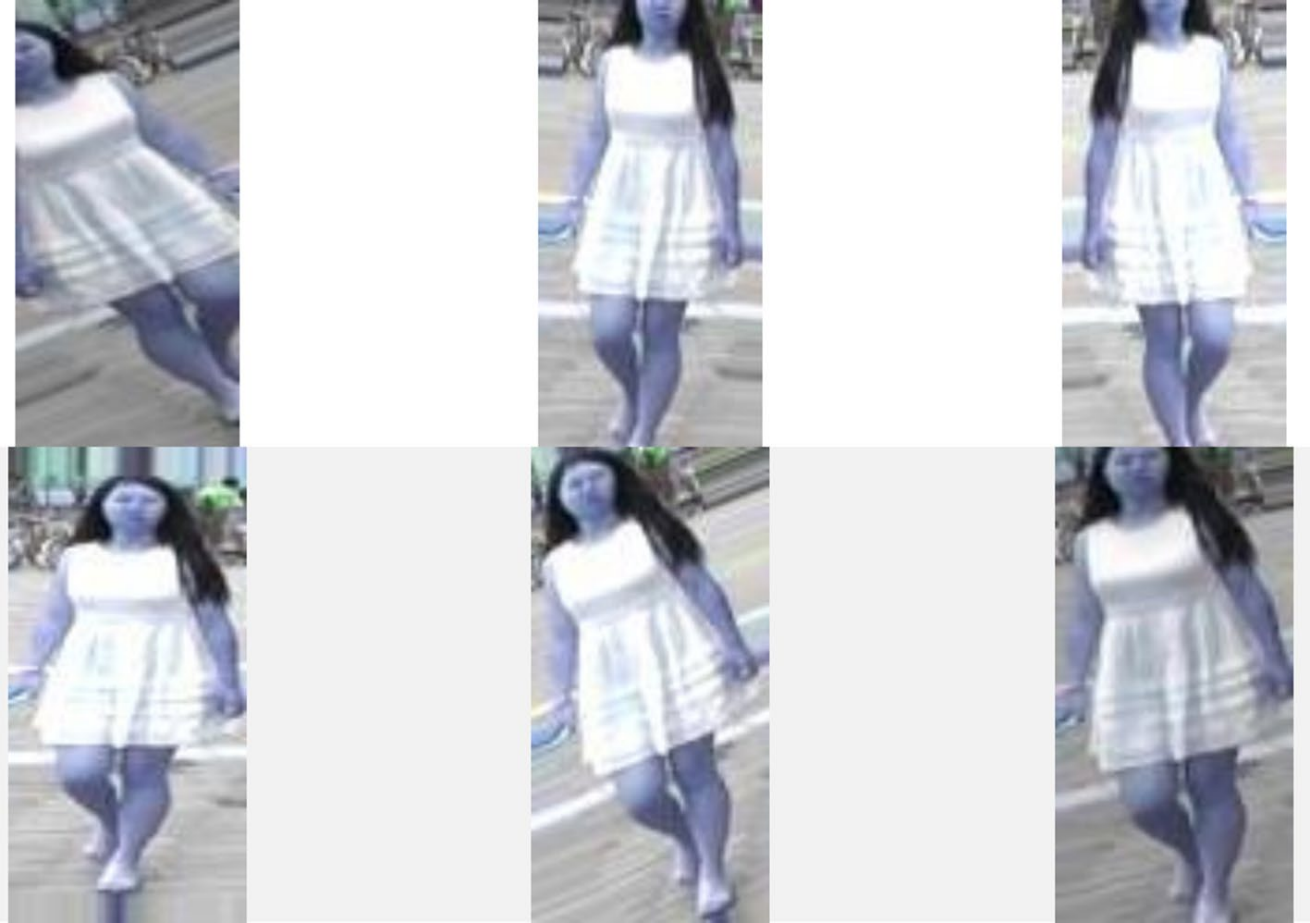
Resulted augmentation

- Rotation-range is a value in degrees (0−180), a range within which to randomly rotate pictures.
- Shear-range is for randomly applying shearing transformations.
- Zoom-range is for randomly zooming inside pictures.
- Horizontal-flip is for randomly flipping half of the images horizontally –relevant when there are no assumptions of horizontal asymmetry.
- Brightness-range: Tuple or list of two floats. Range for picking a brightness shift value from.

for an image in the Fig. 12:

the resulted augmentation is shown in Fig. 13:

So the training dataset will be increased six times.

### Performance analysis

Performance was validated using Market dataset, we have choose samples from the dataset to work on. For 200 persons in recognition, training dataset was increased by augmentation to 21557 images and validation accuracy has remarkable increased to reach 96.23

For 800 person, the accuracy was 93.62% without image augmentation. Image augmentation for 800 persons was not applied because memory issue, since 800 persons are represented by more than 11000 images (more than 66000 images with augmentation).

Comparison of applied augmentation Machine Learning models is in Table 5.

**Table 5:** Results comparison

Without IA	With IA (%)	Notes
82	96.23	200 persons recognition
93.62	NA	800 persons recognition
		(With IA is Not Available due to memory)
		issue because of huge amount of data
		for 800 persons images with augmentation

Parameters were selected depending on some goals. For each parameters, two main goals were considered:

- High accuracy.
- Speed of convergence.

So we can summarize the main advantage of this work as follows:

- Image augmentation for gait recognition was applied with CNN algorithm.
- Best CNN parameters.
- Best CNN design and structure.

The proposed model consists of typical CNN layers with some specific properties:

- First pooling layer with max function as pooling type
- Rest pooling layers in CNN structures with average function as pooling type.
- Batch normalization after every pooling layer.
- Convolution layers with regularizer L2.

After selection best parameters for our proposed model, image augmentation techniques were implemented to make the final model robust as possible.

Comparing to [10] in selected hyper parameters

Ours: LR:8e−4, Epochs:50, Batch size: 10, Optimizer: Adam.

Them: LR:1e−3, Epochs:50, Batch size: 20, Optimizer: Adam.

The average recognition rate reaches 97%, while in ours it reaches 96.23.

There are not many studies with image augmentation to compare with. In addition, No studies are worked on Market dataset.

## Conclusion and future scope

In summary, This work proposed simple and robust model for person recognition using gait model features based on CNN algorithm, this model resulted with edits in the design of CNN model and choosing hyper parameters for some parts of CNN model. This study also introduce Image augmentation in recognition; that helps to make the simple models robust to some changes in images of persons, it generate images of the same frame or view with different conditions. The final model was validated and it performed well and better than background studies.

We can think about some points:

- Improve person recognition by rebuilding implemented methods can be rebuilt with other batch normalization modes and with some pre-processing steps for data-set.
- Using genetic algorithm for some parameters; it needs more processing time and high performance processors.
- Search deeply in Fully connected layer to figure out the validity of changing activation function, or manually select activation function.
- For IA, new conditions can be added to generate more images that handling other variation in images.

The main effective scope is to implement it in real time system for real useful goals.

## Data Availability

The data set is available to public and can be found in https://drive.google.com/file/d/0B8-rUzbwVRk0c054eEozWG9COHM/view.

## Abbreviations

CNN: Convolution Neural Network
SVM: Support Vector Machine
NN: neural network
PCA: Principal Component Analysis
PRGM: Person Recognition based on Gait Model
